# Comprehensive treatment of dementia with Lewy bodies

**DOI:** 10.1186/s13195-015-0128-z

**Published:** 2015-05-29

**Authors:** Brendon P Boot

**Affiliations:** Department of Neurology, Brigham and Women’s Hospital, 221 Longwood Avenue, Boston, MA 02115 USA; Harvard Medical School, 25 Shattuck Street, Boston, MA 02115 USA

## Abstract

Dementia with Lewy bodies is an under-recognized disease; it is responsible for up to 20 % of all dementia cases. Accurate diagnosis is essential because the management of dementia with Lewy bodies is more complex than many neurodegenerative diseases. This is because alpha-synuclein, the pathological protein responsible for dementia with Lewy bodies (and Parkinson’s disease), produces symptoms in multiple domains. By dividing the symptoms into cognitive, neuropsychiatric, movement, autonomic, and sleep categories, a comprehensive treatment strategy can be achieved. Management decisions are complex, since the treatment of one set of symptoms can cause complications in other symptom domains. Nevertheless, a comprehensive treatment program can greatly improve the patient’s quality of life, but does not alter the progression of disease. Cholinesterase inhibitors are effective for cognitive and neuropsychiatric symptoms; rivastigmine has the widest evidence base. Special care needs to be taken to avoid potentially fatal idiopathic reactions to neuroleptic medications; these should be used for short periods only when absolutely necessary and when alternative treatments have failed. Pimavanserin, a selective serotonin 5-HT2A inverse agonist, holds promise as an alternative therapy for synuclein-associated psychosis. Levodopa/carbidopa treatment of parkinsonism is often limited by dopa-induced exacerbations of neuropsychiatric and cognitive symptoms. Autonomic symptoms are under-recognized complications of synucleinopathy. Constipation, urinary symptoms and postural hypotension respond to standard medications. Rapid eye movement sleep behavior disorder is highly specific (98 %) to the synucleinopathies. Nonpharmacological treatments, melatonin and clonazepam are all effective.

## Introduction

Dementia with Lewy bodies (DLB) is an under-recognized disease. The diagnostic criteria have low sensitivity (12 to 32 %) and high specificity (>95 %) [1], so many cases are not diagnosed. Therefore, meta-analytic studies suggesting that DLB accounts for 4 % of dementia diagnoses [2] underestimate the true prevalence [3], which may be closer to 20 % of dementia [4, 5]. Parkinson’s disease dementia (PDD) accounts for a further 3 to 5 % of dementia cases [5, 6]. Both DLB and PDD are due to the pathological accumulation of alpha-synuclein, but patients with parkinsonism for 1 year prior to cognitive decline are classified as PDD [4]. Cognitive decline and parkinsonism are insidious, so the distinction can be difficult to draw and may be influenced by the subspecialty interest of the diagnosing neurologist (for example, movement disorder versus behavioral neurology) [1, 7]. Data on the relative frequency of DLB and PDD may be similarly affected by this subspecialty referral pattern. Whether or not the distinction has treatment implications is difficult to determine. This review highlights the distinction only where clinically relevant differences in outcomes have been noted. Restricting this review only to evidence-based treatments would mean that it could not be comprehensive, since many treatments have not been studied in DLB or PDD populations. In these instances, evidence from Parkinson’s disease (PD) studies is used to guide clinical recommendations.

The management of DLB is replete with quandaries: in choosing to treat one symptom, we often produce complications in other facets of the disease. For example, dopamine replacement for motor symptoms frequently exacerbates a patient’s neuropsychiatric symptoms, antipsychotic treatment of hallucinations risks a potentially fatal adverse reaction, and cholinesterase inhibitor treatment of cognitive symptoms can complicate cardiac and gastrointestinal dysautonomia. These quandaries make the treatment of DLB challenging, yet just as rewarding as navigating between Scylla and Charybdis. The side-effect proclivity applies to medications prescribed by other physicians, so it is essential to rationalize treatment and to communicate with other care providers about the complexities of the disease. An understanding of the pervasive effects of alpha-synuclein can also assist the physician to make sense of nonspecific or vague complaints, particularly when advanced disease impairs a patient’s ability to communicate. For example, a feeling of general malaise or lethargy can reflect orthostatic hypotension, a common and under-recognized feature of the disease.

Many symptoms of DLB are noncognitive in nature, and many are under-recognized [8–10]. It can be helpful to divide the array of symptoms into five symptom categories: cognitive, neuropsychiatric, movement, autonomic, and sleep. Patients often view DLB as a purely cognitive disease, and consequently will not volunteer noncognitive symptoms since they do not believe they are a consequence of the disease. Directed questions in each of the five categories can form the basis of a comprehensive treatment strategy that can improve the patient’s quality of life [11]. The disease course can be rapid, although prognosis varies between individuals. In one study, life expectancy at diagnosis is 2.3 years shorter for DLB compared with Alzheimer’s disease [10].

## Cognitive symptoms

DLB-associated deficits in attention, executive function, and visuospatial ability respond well to cholinesterase inhibitor treatment [12–14]. In meta-analyses, the standardized mean treatment effects are 0.34 for cognition and 0.20 on behavioral and functional measures [13], although most of the source data are from PDD patients. These effects compare favorably with cholinesterase inhibitor treatment of Alzheimer’s disease [15], because the targets of therapy are relatively preserved in DLB. Compared with Alzheimer’s patients, DLB patients have relatively little neuronal loss but profound cholinergic dysfunction [5, 16]. The characteristic fluctuations in cognition in DLB are difficult to manage; they may have multiple contributing causes [17]. Cholinesterase inhibitor treatment is associated with reduced mortality; mortality odds ratios in treatment trials are 0.28 (*P* = 0.03) despite increases in adverse events on therapy (odds ratio 1.64, *P* = 0.0003) [14].

There are no head-to-head trials comparing efficacy of the cholinesterase inhibitors in DLB or PDD, but rivastigmine has the widest evidence base [12, 18, 19]. A Movement Disorder Society evidence-based review concluded that rivastigmine is effective in PDD, but that the data for other cholinesterase inhibitors and memantine are inconclusive [20]. Special care is needed when starting cholinesterase inhibitors in patients with DLB because of frequent disease-associated autonomic dysfunction [21]. Cardiac denervation [22] might increase the risk of symptomatic bradycardia [13, 23], or QT prolongation. Gastrointestinal dysautonomia [24, 25] means patients may be more at risk for the common cholinesterase inhibitor side effects of nausea, vomiting, diarrhea, anorexia, and weight loss [13, 26]. To treat these, the clinician should eschew antagonists of dopamine, acetylcholine, or histamine in favor of 5HT-3 receptor antagonists such as ondansetron or granisetron. Rivastigmine has a transdermal formulation with fewer gastrointestinal side effects. DLB patients frequently have disordered sleep and so may be more likely to experience the vivid dreams that are an under-recognized side effect of cholinesterase inhibitors. These dreams can be limited by avoiding night-time doses; for cholinesterase inhibitors that require twice-daily dosing, the second dose may be given in the afternoon.

Only one of four trials of memantine in DLB/PD/PDD found significant effects in the primary outcome [27]. The results may be driven by data from PDD rather than DLB subjects, and may only be present with concurrent cholinesterase inhibitor treatment [27, 28]. Atomexetine [29], rasagiline [30] and levodopa [31, 32] have variable effects on cognition in PD/PDD populations [33].

## Neuropsychiatric symptoms

### Anxiety and depression

Anxiety and depression are common in DLB; they affect 27 % and 59 % of cases, respectively [10]. Like many nonmotor symptoms of synucleinopathy, anxiety and depression can predate the onset of parkinsonism and dementia by decades [10, 21, 34], possibly due to early pathology in serotonergic projection cells of the dorsal raphe [35, 36]. Incipient synucleinopathy should therefore be considered in the differential diagnosis of late-onset anxiety and depression, particularly in patients without an obvious precipitant and/or subtle parkinsonism [37, 38].

There are no controlled trials of treatments for anxiety in DLB or PDD [33]. Depression was one of four neuropsychiatric symptoms in a composite measure that improved in trials of rivastigmine [39] and olanzapine [27, 40]. A single, uncontrolled trial of citalopram and risperidone in 31 patients found no improvement for either drug after 12 weeks [41]. Otherwise, there is a paucity of evidence in the treatment of depression in DLB. Serotonin and serotonin/norepinephrine reuptake inhibitor antidepressants have mixed results in the treatment of PD-related depression [20, 42, 43]. Electroconvulsive treatment and transcranial magnetic stimulation are both effective in DLB [44, 45].

### Hallucinations and delusions

Hallucinations occur in 60 to 70 % of DLB patients [8]. Commonly, they begin in the first 2 or 3 years of the disease whereas they are a late phenomenon in Alzheimer’s disease [46]. There may also be qualitative differences. In Alzheimer’s disease, hallucinations generally have a threatening or fearful quality [47]. They are often accompanied by delusions of suspicion [47]. In contrast, the hallucinations in DLB are often (but not always) nonthreatening misperceptions of ambiguous stimuli. For example, a patient may misinterpret a shadow to be a person or an animal. Tests of these misperceptions, termed pareidolias, accurately differentiate DLB from Alzheimer’s disease [48]. Electing not to treat these symptoms is often appropriate, but cholinesterase inhibitor therapy is safe and effective [39]. In one study of PDD, more than 90 % of patients reported reduced visual hallucinations with cholinesterase inhibitor use [12]. The hallucinations can be minimized by regular vision correction and a bright light or no light policy, whilst minimizing the risk of falls. Medications that can exacerbate neuropsychiatric symptoms in DLB should also be stopped. These include anticholinergic medications, amantadine, dopamine agonists, monoamine oxidase inhibitors, catechol-*O*-methyl transferase inhibitors, and levodopa, bearing in mind that abrupt cessation can trigger the neuroleptic malignant syndrome [49].

The most fraught decision in the management of DLB relates to the use of antipsychotic medications. In Alzheimer’s disease and all-cause dementia studies, antipsychotic medications are rarely effective at reducing symptoms [50] and they increase the risk of stroke and sudden cardiac death by at least 50 % [51, 52]. Ceasing these medications decreases the risk of death by the same amount [53]. The latter study counters the idea that the antipsychotic-associated mortality is due to confounding by indication. This theory states that end-stage dementia causes increased mortality and also causes the prescription of antipsychotics. Data from these pivotal studies led to a US Food and Drug Administration black-box warning for antipsychotic medication use in dementia and a substantial change in prescribing practice.

A major factor in the success or otherwise of treatment trials of antipsychotic medications in DLB and other dementia relates to the target symptoms. Hallucinations and delusions are more likely to respond to these medications than behavioral disturbance, as described below. Physicians should reserve the use of neuroleptics for symptoms likely to respond to their use, after focused behavioral interventions have been attempted. There is ample evidence of efficacy for these behavioral interventions [54], but limited funds for implementation. Gitlin and colleagues provide an excellent description of such a program [55].

A continued role for antipsychotic treatment remains for the short-term treatment of subjects at risk of harm due to their psychosis [47]. Higher scores on the Neuropsychiatric Inventory may indicate a patient is more likely to respond to the treatment, particularly if the findings are within the domains most amenable to treatment with neuroleptic medication (see discussion below) [56].

DLB patients are particularly at risk of antipsychotic medication morbidity and mortality. Severe neuroleptic sensitivity occurs in 30 to 50 % of patients [57]. Typical antipsychotics (for example, haloperidol) are best avoided [57], but reactions can occur after any neuroleptic and no differences in mortality were found between the atypical antipsychotics in all-dementia clinical trials [50]. Profound sedation, confusion, exacerbations of parkinsonism, rigidity, dysautonomia, and death can occur [4, 16, 57, 58] even after a single dose [57, 59, 60]. These effects are associated with a threefold increase in stroke occurrence and a twofold to fourfold increase in the rate of cognitive decline [50, 53]. There is also evidence to the contrary, however. Antipsychotic medications have been well tolerated in DLB trials [61, 62], and a large observational study that controlled for cardiovascular risk and psychosis severity found no increase in mortality amongst Alzheimer’s disease patients [63]. Irrespective of the controversy over the degree of risk, the large placebo response seen in trials of antipsychotics [27, 50] and the data showing improved survival for those taken off long-term antipsychotics treatment [53] dictate that all new prescriptions of antipsychotics should include a programmed trial of cessation [64].

The choice of which antipsychotic to use is also a vexed question. Quetiapine and clozapine are equally effective in head-to-head PDD trials [49, 65], although other data show mixed results for quetiapine in underpowered trials of DLB/PDD [20, 61, 62, 66, 67]. Despite the paucity of evidence for its efficacy, many clinicians use quetiapine, reserving clozapine for second-line or third-line treatment because of its potential to cause agranulocytosis [68–72]. More data are sorely needed to inform antipsychotic medication choice in DLB. In the interim, it is reasonable to select antipsychotic medications on the basis of their side-effect profiles. Patients at risk for diabetes or hyperlipidemia should avoid quetiapine, olanzapine and clozapine, whereas those with elevated cerebrovascular risk should avoid olanzapine and risperidone [47]. Olanzapine is also associated with motor decline in PD patients with psychosis [20]. The Movement Disorders Society advises against olanzapine use in PD [20], advice that might reasonably be extended to DLB patients. Irrespective of which antipsychotic medication is used, prescribers should be wary of the cardiac denervation seen in the synucleinopathies [22] and so consider monitoring the QT interval. This is especially important when cholinesterase inhibitor and neuroleptic medications are used together. Fortunately, several new agents without such side effects are under development. For example, a recent trial of pimavanserin, a selective serotonin 5-HT2A inverse agonist, shows great promise in the treatment of PDD, both in terms of treatment response and trial design [73].

### Agitation and behavioral disturbance

Agitation and behavioral disturbance often respond to simple measures such as caregiver training, removal of fear triggers, and increased social interaction [74]. Many triggers for agitation are fleeting, and episodes of agitation are self-limiting, so watchful waiting is often preferable to antipsychotic prescription [53]. In the late stages of disease, when patients have difficulty expressing their needs, pain is often a trigger for agitation: investigation for potential sources of pain and empiric treatment with simple analgesics such as acetaminophen should be first-line therapy [75]. Antipsychotic medications have the same qualifications to their use as noted above. Furthermore, behavioral disturbances such as sleep–wake cycle disturbance, shouting, oppositional behavior, pacing, agitation, and aggression are not good targets of therapy for the neuroleptic medications. There are numerous carer training programs designed to decrease the disturbances, but only six medications have evidence for efficacy, three of which are readily accessible [76–78]. Successful programs train caregivers to understand care situations from the perspective of people with moderate to severe dementia, and to adapt their approach to such encounters to encourage respect for the patient’s personhood [55]. Extensive training is required for program success [76–78].

## Movement symptoms

The motor symptoms and signs of DLB are similar to those found in PD, including rigidity, bradykinesia, tremor, and gait difficulties. They may respond to physical therapy and home safety modification. As is the case in PD, the primary prevention of falls is paramount in DLB. Education on the importance of this point can be punctuated with advice such as ‘gravity is your mortal enemy’. Repeated falls should trigger a rapid assessment and treatment of the cause(s).

The same medications that are used in PD for movement symptoms are used in DLB, but they are usually less effective than in PD [79]. Their use is often limited because of their tendency to exacerbate the neuropsychiatric features of DLB [79–81]. Levodopa/carbidopa is most useful in patients with prominent parkinsonism and few or no neuropsychiatric symptoms [81]. Levodopa/carbidopa is used in preference to the dopamine agonists because the latter are more likely to induce compulsive behavior. In one series, 24 % of PD patients taking dopamine agonists suffered from this potentially devastating constellation of side effects [82]. Amantadine may reduce the severity of the compulsive behaviors, but can also worsen dysautonomia and hallucinations [20].

Physical therapy and home modification are effective in PD. Cholinesterase inhibitors may exacerbate tremor, but only mildly so, and do not otherwise worsen parkinsonism [26, 39]. Where possible, medications that can induce parkinsonism should be avoided – these include the dopamine receptor blocking anti-emetics (for example, prochlorperazine and metoclopramide) and neuroleptics.

## Autonomic symptoms

### Constipation

The most common autonomic complications of synucleinopathy are under-recognized: 89 % of PD patients have constipation or diarrhea. Sixteen percent of the patients have been hospitalized for bowel obstruction [24, 25]. Patients may be unaware that this is a consequence of their DLB, since the symptoms usually predate other aspects of the syndrome by many years [10, 21, 34]. Directed questions and early treatment with a high-fiber diet, exercise, stool softeners, psyllium [83], polyethylene glycol [84], methylcellulose, docusate, and misoprostol [49] are effective. Increased bowel activity is a common (and in this case welcome) side effect of cholinesterase inhibitor therapy.

### Genitourinary symptoms

Up to 83 % of PD patients experience urinary frequency, urgency, and incontinence [85], for which oral trospium and transdermal oxybutyinin are effective [20] alternatives to trihexyphenidyl and oral oxybutyinin, which cause confusion [8, 86–88]. Tamsulosin and bethanol chloride are effective for prostatism and urinary retention [49]. Sildenafil is effective for erectile dysfunction in PD [89], but prescription requests should trigger a review to determine whether levodopa or dopamine agonist treatment is inducing hypersexual behavior [82, 85].

### Postural hypotension

Orthostatic symptoms are common in DLB [58, 90]; their frequency and severity is a strong predictor of prognosis [91]. Patients may not describe classic postural symptoms, but instead mention nonspecific weakness or lethargy. Reduction or cessation of antihypertensive medication, meal fragmentation, salt liberalization/supplementation and compression stockings [20], fludrocortisone [92], and domperidone [92] are all effective in the synucleinopathies. Cholinesterase inhibition [93] and pyrodostigmine [94] also improve these symptoms.

## Sleep symptoms

### Excessive daytime sleepiness

It is easy to underestimate the effect that excessive sleepiness can have on a patient’s quality of life. Sedating medications should be ceased and obstructive sleep apnea, primary sleep disorders, and nocturia should be ruled out. Caffeine is a useful treatment in those without periodic leg movement disorder of sleep or restless leg syndrome [95]. The evidence for methylphenidate and dextroamphetamine is mixed [20]. Modafinil was effective in two of three PD trials [20, 96]. Eighteen of 20 patients responded well in a small, unblinded trial of armodafinil [97].

### Rapid eye movement sleep behavior disorder

Seventy-six percent of DLB patients act out their dreams [98]. When confirmed by polysomnography, rapid eye movement sleep behavior disorder is 98 % specific to the disorders of synuclein [99]. The disorder may not require treatment unless it induces excessive daytime sleepiness, or poses a physical risk to the patient or their bed partner. Simple instructions can prevent harm: remove sharp objects from the bedside, use soft barriers around the bed, or sleep in a tightly closed sleeping bag (cocooning) [95, 100]. The enacted dream often involves being chased or attacked, so bed partners should avoid the dreamer lest they be incorporated into the dream and attacked [95]. Randomized controlled trials demonstrate that melatonin [101–103], rivastigmine [104], and bed alarms that play soothing messages from caregivers [100] are effective rapid eye movement sleep behavior disorder treatments. The short half-life of melatonin means that a second dose may be needed if sleep is interrupted. Low-dose clonazepam is a recommended second-line therapy [105].

### Restless leg syndrome and periodic limb movement disorder

These conditions often accompany the disorders of synuclein, and each other [106]. The treatment options are identical for the two disorders; however, periodic limb movement disorder does not require treatment unless it is disrupting sleep or sleep architecture. There are no trials of restless leg syndrome or periodic limb movement disorder treatment in the context of DLB. The dopamine agonists are not recommended in this setting for the reasons outlined earlier. Standard treatment of restless leg syndrome and limb movement disorder with carbidopa/levodopa, benzodiazepines (and especially clonazepam), and the alpha-2-delta calcium channel ligands (gabapentin, gabapentin enacarbil, and pregabalin) are effective in PD patients [106].

## Conclusions

DLB is a complex disease with many challenging treatment decisions. It is often under-recognized in the clinic, in part because the core clinical diagnostic features – fluctuations in cognition, visual hallucinations, and parkinsonism – are nonspecific and subject to varied interpretation. For example, how much parkinsonism is enough to qualify for this feature? Treatment options can improve quality of life, but do not alter the course of the disease. For many symptoms, the best treatments are nondrug treatments. Regular reviews aimed at rationalizing therapy can be beneficial. For example, anti-hypertensive medications that were erstwhile well tolerated may induce postural dizziness because alpha-synucleinopathy weakens neurovascular tone. The most difficult decision relates to the use of antipsychotic medications; these occasionally benefit patients with hallucinations and delusions, but severe reactions such as prolonged rigidity and decreased responsiveness are common in DLB. Recent trials of antipsychotics with novel mechanisms of action hold promise for this vulnerable population.

## Note

 This article is part of a series on *Lewy Body Dementia*, edited by Ian McKeith and James Galvin. Other articles in this series can be found at http://alzres.com/series/LewyBodyDementia

## Abbreviations

DLB: dementia with Lewy bodies
PD: Parkinson’s disease
PDD: Parkinson’s disease dementia
